# Ejecting chick cheats: a changing paradigm?

**DOI:** 10.1186/1742-9994-8-14

**Published:** 2011-06-13

**Authors:** Tomáš Grim

**Affiliations:** 1 Department of Zoology and Laboratory of Ornithology, Palacký University, tř. Svobody 26, 77146 Olomouc, Czech Republic

## Abstract

Evolutionary arms-races between avian brood parasites and their hosts have typically resulted in some spectacular adaptations, namely remarkable host ability to recognize and reject alien eggs and, in turn, sophisticated parasite egg mimicry. In a striking contrast to hosts sometimes rejecting even highly mimetic eggs, the same species typically fail to discriminate against highly dissimilar parasite chicks. Understanding of this enigma is still hampered by the rarity of empirical tests - and consequently evidence - for chick discrimination. Recent work on Australian host-parasite systems (*Gerygone *hosts vs. *Chalcites *parasites), increased not only the diversity of hosts showing chick discrimination, but also discovered an entirely novel host behavioural adaptation. The hosts do not desert parasite chicks (as in all previously reported empirical work) but physically remove living parasites from their nests. Here, I briefly discuss these exciting findings and put them in the context of recent empirical and theoretical work on parasite chick discrimination. Finally, I review factors responsible for a relatively slow progress in this research area and suggest most promising avenues for future research.

## Introduction

Science advances by a triad of major types of contributions: most frequently by collecting data to test hypotheses that were already formulated or tested by others (most papers in any scientific journal), less often by suggesting novel hypotheses (e.g. [1]) and most rarely by discovering completely new and unexpected natural phenomena (e.g. [2]). Recent work by Sato et al. [3] and Tokue and Ueda [4] belong to this last category of scientific endeavour. These two studies showed for the first time that hosts of parasitic birds (genus *Gerygone*) may defend against alien (little bronze-cuckoo *Chalcites minutillus*) chicks by *physically removing the living parasite *from the nest, so saving the lives of their own nestlings. This qualitatively novel host behaviour contrasts with host deserting [5], feeding less [6] or pecking [7] parasite chicks which were the only anti-chick defences documented so far. Coupled with other recent studies of parasite chick discrimination [7,8], these findings have important implications for our understanding of host-parasite relationships specifically and parent-offspring interactions in general.

## A novel host anti-parasite adaptation

Interactions between brood parasites and their hosts are one of the most intensely studied natural models of coevolution, recognition and learning [9-11]. Until recently, the majority view of host defences to mitigate costs of parasitism was that most host species reject at least some proportion of alien eggs but that hosts typically accept alien chicks (reviewed in [12-14]). Virtually any paper or textbook on brood parasitism is a testimony of this (for an exception see [15]). Some studies even claimed 'hosts do not reject nestlings' [16] despite rare but existing evidence to the contrary (see older references in Table one in [12]). This view prevailed for decades despite rarity of any thorough studies on host responses to alien nestlings [13]. Further, theoretical arguments seemed to explain an apparent absence of chick discrimination. An elegant and influential model by A. Lotem [17] showed that imprinting on the first *clutch/egg *is adaptive in hosts of evicting parasites whereas imprinting on the first *brood/chick *is maladaptive. However, the model was based on a critical assumption of *learned *recognition with naïve breeders being unable to reject the parasite by definition (thus, the model does not hold for non-learned innate recognition, see p. 744 in [17]). That assumption does not hold empirically for many hosts because already first-year naïve breeders often reject parasitism and no effects of female age on her ability to recognize parasite eggs were typically found [e.g. [18-20], Grim, Samaš, Cassey and Hauber in prep.] (this, of course, does not exclude an age-related fine-tuning of innate or learned discrimination abilities in other host species [21]). Also, Lotem's model [17] is unlikely to apply in hosts that show ~100 rejection of alien eggs, i.e., there is no inter-individual, and by implication intra-individual variation in host behaviour [22,23]. Limited data for parasite chick discrimination in relation to host individual experience [5,6] also suggest that the misimprinting model does not apply to chick discrimination in the host-parasite systems studied so far (but see [7] for a case of *conspecific *parasites). As the known biological reality of inter-specific parasites does not generally fit assumptions of the Lotem's model [17], the misimprinting argument does not constrain chick discrimination [5,6,12,24-26].

Brood parasitism literature was always heavily biased in favour of egg discrimination [13]. However, absence of evidence is not evidence of absence and, strikingly, all recent papers that looked for parasite chick discrimination by hosts have found some evidence for host differential responses to alien nestlings ([3-5,7,27,28]; see also p. 750 in [29] and [30]; earlier studies reviewed in [12,31]). Although there are many studies based on nests with parasite chicks, they typically did not follow parasite chicks up till fledging. Such studies are inconclusive because discrimination can occur shortly before [27,31] or even after fledging [32]. There are few careful studies showing that chick rejection does not occur [9]. So, why are the new studies [3,4] so exciting?

The most striking aspect of the newly described chick discrimination systems [3,4] is that chicks were not simply abandoned (as in the previous studies documenting chick discrimination under natural conditions [12]). Instead, hosts grasped the parasite nestlings with their beaks and ejected them from their nests. Although eviction of dead chicks is known as an expression of nest-cleaning behaviour in many birds (discussed in [12]), such behaviour was never previously observed in the context of host-parasite interactions.

Tokue and Ueda [4] work is further exciting for another reason. Previously reported chick discrimination host responses were highly costly to hosts in terms of (a) prolonged care for the parasite chick before its desertion [33], (b) high rates of rejection errors [3], or (c) a complete loss of host own progeny ([5]; but see [7] for a case study of *conspecific *brood parasites). In contrast, Tokue and Ueda [4] showed that chick discrimination may take place (a) almost immediately after the parasite hatches, and (b) without rejection errors (though in small sample sizes), thus (c) rejecting host individuals saved their whole current parental investment before the parasite chick had a chance to destroy it.

Supplementary video materials to both Sato et al. [3] and Tokue and Ueda [4] clearly demonstrate that such a physical task is feasible even for small hosts like *Gerygone *warblers. As clear terminology is essential to science, I suggest the removal of alien chicks is referred to not as 'eviction' (as in [3]) but as 'ejection'. By definition, *eviction *is performed by a cuckoo chick when pushing host eggs and/or nestlings from a host nest (see [9,34,35] and references therein). In contrast, *ejection *is performed by a host parent when removing parasite eggs (or nestlings) from its nest [9].

Admittedly, sample sizes were limited in both new chick ejection studies. Still, in a Popperian vein, even a single observation of hosts rejecting an alien chick falsifies the hypothesis that hosts always accept them. Of course, as in the egg discrimination studies, we need to consider a possibility of chick recognition errors (as documented by Sato et al. [3]). However, such errors themselves suggest that hosts respond to chick own-vs.-parasite identity. Also, we need more detailed data showing that ejected nestlings did not suffer from a disease - birds are known to remove sick and/or dead chicks from their nests (see references in [12]).

Another aspect of the two *Gerygone*-*Chalcites *studies that should be highlighted is methodological. Both papers provided readers with *direct *video-recording evidence of chick discrimination (see the supplementary on-line video files for both studies). No paper on egg discrimination has provided readers with such direct evidence perhaps because some journals are reluctant to publish electronic supplementary files. Still, various journals did publish supplementary video files for brood parasitism studies but solely for studies of parasitic chicks [2-4,7,36]. Perhaps journal editors and reviewers do not encourage it because egg discrimination is a well-known phenomenon, unlike chick discrimination. In the future, such video-recording evidence might be published as a useful supplementary material in egg discrimination studies too.

Egg discrimination studies often lack controls (see, e.g., the three published studies with the highest sample size: [28,37,38]). Specifically, egg discrimination studies did not always include control unmanipulated nests (to quantify background nest desertion rates that are not a response to parasitism) and/or observed nests directly (to exclude a risk that a parasite egg disappeared not by host ejection but by being removed by predators, brood parasites, or conspecifics). Both Sato et al. [3] and Tokue and Ueda [4] employed control nests for comparisons and monitored nests by continuous video-recording. This might inspire future egg discrimination studies to rest on more robust methodologies than before.

The present arguments are not meant to devalue the egg studies in any way - only by pinpointing imperfections of our past research we can improve it in the future. This holds for studies of both eggs and chicks. Further, research at the chick stage should not constrain but *complement *research at the egg stage. Overall, I explicitly stress the importance of balanced empirical and theoretical research at all developmental stages, i.e., eggs, chicks and adults [28].

## New findings fit theory

From a theoretical point of view it is remarkable that both systems fit predictions from theory. Both the 'rarer enemy effect' [12], an extension of rare enemy effect, and the 'strategy-blocking' models [25,26] predict that chick discrimination should evolve mostly in hosts that do *not *successfully reject *natural *cuckoo parasitism (i.e., hosts are either acceptors of any, even highly dissimilar foreign eggs, or their existing rejection abilities are compromised by evolved cuckoo egg mimicry). This is essentially because of the asymmetry of cost/benefit ratios of egg vs. chick discrimination (chick discrimination requires stronger selection pressure to evolve than egg discrimination [12]) and an inevitable fact that the parasite is always present in a host nest earlier as an egg and only later in the form of a nestling. Obviously, a host correctly rejecting parasite eggs ('rare enemies') will have no chance to face parasite chicks (which, as a consequence of *host own *behaviour, become even '*rarer *enemies'). Paradoxically, egg-rejecting hosts themselves eliminate selection pressure that would enable them to evolve an ability to discriminate parasite chicks [12]. Thus, successful previous lines of defence (including nest defence [39]) decrease positive selection pressure on later lines of defence. Indeed, gerygones are currently acceptors of *natural *cuckoo eggs ([3], see also Table one in [12]) and, interestingly, recent work [40] suggests that this might not be because of mimicry but because dark cuckoo eggs are cryptic in dark host domed nests. Still, this has the same consequences for evolution of host defences - egg crypsis forces hosts to accept parasitism at the egg stage similar to egg mimicry, phylogenetic constraints and other mechanisms responsible for high similarity in host vs. parasite phenotypes [41].

Given that gerygones are able to eject non-mimetic *experimental *eggs (R. Noske pers. comm.) it is possible that cryptic cuckoo eggs evolved as an evolutionary response to host discrimination simply because dark cryptic eggs would escape visual host discrimination. This novel hypothesis provides an alternative explanation for the evolution of cryptic eggs not considered by Langmore et al. [40]. Evolution of mimetic eggs (similar to host eggs) or cryptic eggs (similar to dark nest interiors) represent two alternative pathways with effectively the same consequences - both cryptic or mimetic egg appearances force hosts to accept parasitism and reduce selection pressure on the evolution of chick discrimination [12,40].

Older empirical data fit the rarer enemy effect well (Table one in [12]) and the strategy-blocking models [25,26] and novel observations by Sato et al. [3] and Tokue and Ueda [4] support the same views (see also [30]). Additionally, low clutch sizes and high risks of multiple parasitism by cuckoos may contribute to chick ejection being a more adaptive strategy than egg ejection in these hosts (the 'egg dilution effect' hypothesis [42]).

Chick ejections from the new studies [3,4] are compatible with both innate [12,26] and learned [17,43] discrimination. Although it is unlikely, due to the short exposure of hosts to their own chicks, the latter option cannot at present be safely evaluated because the ejected cuckoo chicks hatched shortly *after *the host chicks (see also [7] and a discussion of 'sibling imprinting' in [24]). Only one study [44] followed individuals of known breeding histories to test the effects of experience on *desertion *of a single parasite vs. a single own chick under natural unmanipulated conditions in the superb fairy-wrens *Malurus cyaneus*. Nothing is known about parental age effects on chick discrimination (and, by implication, about innate and learned bases of such discrimination) in both chick-*ejecting *gerygones and any other study systems with presumed or documented defence against nestling parasites (but see a laboratory study [6]). Long-term experimental studies with individually marked naïve first-time breeders with subsequently *manipulated *breeding histories (cf. natural un-manipulated observations in [44]) are needed to see whether and how chick discrimination ability changes during an individual's lifetime.

It remains to be seen whether the prevalence of parasite chick ejection extends to other gerygone congeners (that are often victimized by *Chalcites *cuckoos). If so, the genus *Gerygone *may become an excellent model for the study of parasite-host coevolution at the nestling stage.

## Constraints on detecting and studying chick discrimination

What is the general lesson from recent studies on chick discrimination? The traditional conception of host inability to discriminate against divergent nestlings was misleading. Why?

(a) Due to fundamental differences in avian and human vision it makes little sense to look at host vs. parasite phenotypes and judge their similarity [41,45]. All previous studies that claimed dis/similarity of host vs. parasite nestlings were based on subjective human vision-based assessment. Only very recently, the *assumption *of chick differences was tested by objective analyses of what host parents really perceived [8].

(b) Hosts may pay attention only to a specific trait (e.g., gape colour reflectance in UV) and ignore other, perhaps less informative (but for humans more conspicuous!) traits (see also [8,46]). Such mechanisms were already documented in the context of egg discrimination: some hosts pay attention to UV and blue colours but ignore other regions of egg reflectance spectra [45] and discriminate alien eggs based on blunt - but not sharp - egg pole appearance [47,48].

(c) In contrast to egg discrimination, which is primarily or solely a visual task (which fits human sensory biases), the discrimination of chick impostors might be based on single or combined visual, acoustical or even olfactory cues (that are harder to detect in the vision-dominated human sensory world [5,12]). Additionally, I suggest a novel potential cue for chick discrimination: chick behaviour, e.g., posturing or wing-shaking, typically differs between host and parasite chicks [36], and, thus, hosts might base their discrimination on such behavioural traits. Also the eviction behaviour is performed solely by cuckoo nestlings and, consequently, might be used by hosts to identify the parasite [30]. This hypothesis could be tested in host-parasite systems where the behaviour of nestling parasites deviates from that of host nestlings (examples reviewed in [36]). Further, discrimination cues were typically tested in isolation in previous studies (e.g., visual cues only: [46]; acoustical cues only: [49]). I suggest, that a particular cue may not work *per se*, or additively with another cue, but could trigger discrimination only in interaction with other cues (for an example of testing a similar 'interactive' hypothesis see [28]).

(d) Discrimination need not rely on recognition cues - host-parasite chick similarity may be irrelevant for host decisions to accept or reject chicks. Recognition-free discrimination mechanisms rest on predictable differences in non-phenotypic cues associated with the presence of a parasite chick in the host nest. Such external cues include brood size [3], amounts of care in terms of feeding rates [33], or the length of parental care [27]. Except for one work [27], external cues like provisioning costs or temporal costs of care were not experimentally manipulated in any study of chick discrimination.

(e) Chick discrimination may result not only in an outright rejection of the parasite by ejection [3,4], pecking [50], desertion [27] or starvation [51] but may also be manifested in feeding parasites with a lower quality [52] and/or quantity of prey [53]. Consequently, parasite chicks may only decrease in growth instead of dying [46]. This makes potentially *continuous *chick discrimination harder to detect than a *dichotomous *host decision to remove an alien egg or to let it stay in the nest.

(f) Some subtler tactics of chick discrimination happen at temporal scales of days [5] to weeks [33]. Thus, they are much harder to detect than a simple act of egg ejection that occurs at temporal scales of days, hours or even seconds [9].

(g) Chick discrimination may be poorly developed before fledging [31]. Very few studies followed the fates of parasitized nests up to fledging (reviewed in [12,31]; see also [33-35]. Moreover, discrimination may start after fledging [12,32] and we lack detailed info on post-fledging care in most host-parasite systems [9].

(h) That hosts accept parasite chicks under natural conditions does not mean that hosts lack chick discrimination abilities - those might be countered by evolved chick mimicry (just like egg rejecters are sometimes forced to accept alien eggs that are highly mimetic [9,15,22]). Therefore, without experimental manipulation of chick traits [46] or testing host responses to chicks of different species [6] it is not possible to say anything conclusive on the existence or absence of chick discrimination in any host.

On the other hand, not all cases of parasite chicks not thriving in host nests are evidence for specific host anti-chick defences [14,53]. Also, similarities between parasite and host chicks is not evidence of mimicry, or, by implication, of chick discrimination. This is because such similarities may be non-mimetic - they may arise due to multiple reasons unrelated to brood parasitism [41]. Thus, studying mechanisms of chick discrimination provides theoretically, logistically and interpretatively more challenging tasks than the research on egg discrimination does. These eight factors, coupled with the previous rarity of empirical work, may explain the traditional myth of the absence/rarity of chick discrimination.

## Conclusions

Recent years have seen an increasing interest in host responses to parasite chicks (14) and empirical data for chick discrimination has begun to accumulate. This shifting paradigm has already been reflected in some textbooks [10,11]. Where to next?

(a) Various recent studies (see above) showed that just because chick discrimination is not easy to document, does not mean that it is non-existent or unimportant. An emerging picture is that rejection of chick cheats may not be a rare phenomenon. Clearly, more thorough documentation of host abilities to reject alien chicks across different species is needed (see also p. 750 in [29]) as a starting point for future studies of causes and consequences of host chick discrimination. Candidate study systems are plenty [12,28,30,31].

(b) Several studies [7,27,49] confirm experimental tractability of diverse chick discrimination systems. Still, except for a single well studied system [5,8,40,44,49] we know next to nothing about proximate chick phenotype cues that trigger parental discrimination, repeatability and heritability of chick discrimination abilities, or how an ability to discriminate is moulded by experience. Generally, we have no information on what the specific mechanisms of discrimination at the cognitive, neural and physiological levels from any host are. This holds also for egg studies. Further, non-representative sampling across host taxa and low research effort in this study area prevent analyses of phylogenetic patterns of chick discrimination. Better knowledge of the phylogenetic distribution of chick discrimination will enable us to address additional exciting questions, e.g., which life-history host traits facilitate or constrain evolution of host defences against foreign chicks [24] and whether discrimination of alien chicks by particular hosts is a specific co-evolutionary adaptation or 'collateral damage' resulting from general host life-history traits unrelated to parasitism *per se *[28,54].

(c) The successful incorporation of objective measurements of parasite/host phenotypic dissimilarity coupled with realistic modelling of how hosts perceive such dissimilarity [23,45,55-57] was a major advance for brood parasitism studies at the egg stage. However, we still lack studies that would parallel such advances at the nestling stage (but see [8,58]).

(d) An understanding of chick discrimination will be further enhanced if theoretical models [12,17,25,26,43] and empirical data will mutually inform each other.

In the past, the study of brood parasitism at the egg stage was mostly empirical whereas studies at the nestling stage mostly relied on theoretical work [9,17]. Recently, the parasite nestling field went through the discovery stage of scientific work with various cases of chick discrimination being found across the globe. It is exciting to see that future experimental work on brood-parasite host arms-races, as a textbook example of co-evolution, is no longer hindered by the lack of suitable model study systems.

## Competing interests

The author declares that he has no competing interests.
